# Sleep quality and movement behaviors in school-aged children with overweight and obesity: a scoping review

**DOI:** 10.3389/fpubh.2026.1898454

**Published:** 2026-10-01

**Authors:** Juan A. Barahona, Jhon Alex Zeladita-Huaman, Juan Pablo Aparco, Ana Sarmiento-Riveros, Roberto Zegarra-Chapoñan, Héctor Burgos

**Affiliations:** 1 Doctoral Program in Nursing, Faculty of Medicine, Universidad Nacional Mayor de San Marcos, Lima, Peru; 2 School of Nursing, Faculty of Health, Universidad Santo Tomás, Santiago, Chile; 3 Academic Department of Nursing, Faculty of Medicine, Universidad Nacional Mayor de San Marcos, Lima, Peru; 4 Academic Department of Nutrition, Faculty of Medicine, Universidad Nacional Mayor de San Marcos, Lima, Peru; 5 Faculty of Health Sciences, Universidad María Auxiliadora, Lima, Peru; 6 +Salud, Research Center for Prevention and Health Care, Facultad de Salud, Universidad Santo Tomás, Santiago, Chile

**Keywords:** 24-h movement behaviors, childhood obesity, physical activity, school-aged children, sedentary behavior, sleep quality

## Abstract

**Background:**

Sleep quality and movement behaviors are key determinants of child health and play an important role in preventing overweight and obesity. In the Americas, these behaviors coexist with increasing screen exposure and sedentary lifestyles from early ages. However, the available evidence shows methodological heterogeneity and an incomplete assessment of sleep’s multidimensional nature. This scoping review aimed to map the scientific evidence on the relationship between sleep quality and movement behavior profiles in schoolchildren with overweight or obesity in the Americas.

**Methods:**

A scoping review was conducted following the Joanna Briggs Institute methodology and the PRISMA-ScR guidelines. A systematic search was performed in PubMed, Scopus, Web of Science, and LILACS for studies published between 2002 and 2024. Observational and intervention studies examining movement behavior profile and sleep quality in school-aged children with overweight or obesity were included. The evidence was synthesized using descriptive narrative analysis.

**Results:**

A total of 46 studies were included, most of which were conducted in the United States and Canada and predominantly used cross-sectional observational designs. Several studies used objective measures of movement behaviors and standardized sleep assessment tools. Moderate-to-vigorous physical activity (MVPA) was consistently associated with more favorable sleep outcomes, whereas sedentary behavior and nighttime screen exposure were linked to poorer sleep indicators. Important gaps remain in the multidimensional assessment of sleep quality and in understanding how movement behaviors interact within broader obesogenic and self-regulatory processes.

**Conclusion:**

The available evidence indicates that sleep quality, physical activity, and sedentary behavior are closely interconnected in school-aged children with overweight or obesity, forming movement behavior profiles associated with obesity risk. Behavioral self-regulation may represent an important yet insufficiently explored mechanism linking these factors. Future research should prioritize multidimensional sleep assessment and integrated movement-behavior approaches to support more effective school-based interventions and public health strategies across the Americas.

## 1 Introduction

Childhood obesity has become one of the most urgent global public health issues of the 21st century, with serious implications for metabolic health, cardiovascular risk, and overall well-being (1). Over the past forty years, the prevalence of overweight and obesity among children and adolescents has increased sharply worldwide, reflecting complex interactions among biological, behavioral, and environmental factors (2, 3). Beyond its well-known physical health effects, childhood obesity is also linked to psychological, cognitive, and social problems, increasing the risk of chronic illnesses and lowering quality of life throughout life (4). Recently, sleep has gained recognition as a vital modifiable lifestyle factor influencing obesity risk and pediatric health outcomes (5). Emerging evidence shows that insufficient or poor-quality sleep disrupts metabolic regulation, appetite control, and energy balance by affecting hormonal pathways involving leptin, ghrelin, and cortisol (6, 7). In children and teens, inadequate sleep is consistently associated with higher body mass index (BMI), increased fat accumulation, and elevated cardiometabolic risk (5, 8). Additionally, disturbances in sleep patterns—including reduced sleep efficiency, longer sleep latency, and sleep fragmentation—are linked to behavioral and emotional problems that may lead to unhealthy lifestyle choices and behaviors that contribute to obesity (9, 10). Environmental factors, such as excessive sedentary behavioral routines, also contribute to sleep issues among youth (11), although they are not the primary focus of this study. Recent evidence reinforces the need to consider these behaviors collectively. School-based multicomponent interventions addressing physical activity, sedentary behavior, and sleep have shown modest improvements in sedentary behavior and sleep duration, highlighting the interdependence of movement behaviors across the 24 h (12).

Within the emerging 24-h movement behavior paradigm, sleep, MVPA profiles, and sedentary behavior are viewed as interconnected components of daily time use that collectively influence metabolic health and child development (3, 5). In this framework, sleep is increasingly recognized as a crucial element of the 24-h movement behavior model, alongside MVPA profiles and sedentary behaviors, that influences obesity risk and overall health outcomes in children (13).

MVPA profiles are another key behavioral factor for pediatric health and are often included in comprehensive programs aimed at preventing obesity and overweight (14). Regular exercise helps regulate metabolism, reduces body fat, and can improve sleep quality by influencing circadian rhythms, autonomic nervous system function, and thermoregulation (15–17). Conversely, sedentary behaviors—especially prolonged sedentary routines—are associated with poorer sleep, fewer sleep-related behaviors, and a higher risk of obesity in children and adolescents (18). These findings suggest that sleep, MVPA profiles, and sedentary behavior are interconnected factors that may influence one another in shaping childhood obesity and overall health (13, 19). Although research on these relationships is growing, many studies focus solely on sleep-related behavioral patterns, neglecting other dimensions of sleep quality, such as efficiency, latency, regularity, and subjective satisfaction (20). Likewise, while MVPA profiles and sedentary behaviors have been widely studied, fewer investigations have explored how these behaviors interact with multiple dimensions of sleep quality, particularly among children with overweight or obesity (21). Beyond their physiological implications, sleep and movement behaviors may also influence neurobehavioral processes involved in self- and emotion regulation, as well as health-related decision-making. Emerging evidence suggests that poor sleep quality and sedentary lifestyles are associated with impairments in inhibitory control, attentional regulation, and behavioral organization, potentially contributing to obesogenic behavioral patterns and unhealthy lifestyle habits in children and adolescents (22, 23).

This fragmented evidence limits a comprehensive understanding of the complex behavioral pathways linking sleep, movement behaviors, and pediatric obesity, thereby hindering the development of integrated, evidence-based prevention strategies. Although previous reviews have examined isolated associations among sleep duration, physical activity, sedentary behavior, and obesity, the available evidence remains conceptually fragmented in assessing the multidimensional nature of sleep quality and its interactions with MVPA profiles among children with overweight or obesity. Furthermore, substantial heterogeneity persists across study designs, measurement approaches, and conceptual definitions. Consequently, a scoping review was considered the most appropriate methodology to map the breadth of available evidence (24), identify knowledge gaps, and clarify methodological trends across the Americas. However, this previous synthesis was not specifically focused on children with overweight or obesity, leaving an important evidence gap regarding the interaction between multidimensional sleep quality and movement behaviors in this population. Therefore, this review aimed to systematically map and synthesize the scientific evidence on the relationship between sleep quality and MVPA profiles among school-aged children with overweight or obesity, with particular attention to family and school environments, to inform future research and support the development of integrated health promotion strategies.

## 2 Methodology

### 2.1 Study design

The scoping review followed methodological guidance from the Joanna Briggs Institute (JBI) and was reported according to the PRISMA-ScR checklist (24, 25). This approach was selected to systematically map the existing scientific evidence and identify research gaps regarding the relationship between sleep quality and MVPA profiles among schoolchildren with overweight or obesity.

### 2.2 Research question

The research question was developed using the Population–Concept–Context (PCC) framework, which is recommended for scoping reviews. Population (P): schoolchildren and adolescents with overweight or obesity. Concept (C): MVPA profiles and sleep quality. Context (C): educational and family environments. Therefore, the main question for this review was: What scientific evidence exists regarding the relationship between sleep quality and MVPA profiles in schoolchildren with overweight or obesity in the Americas?

### 2.3 Inclusion and exclusion criteria

We included studies that investigated school-aged children and adolescents with overweight or obesity, regardless of sex or sociodemographic background, and assessed both MVPA profiles and sleep-related outcomes using validated qualitative or quantitative instruments. Eligible studies were conducted in educational or family environments and included observational, quantitative, qualitative, or mixed-methods designs. We considered only studies published in English, Spanish, or Portuguese and conducted in countries in the Americas. Studies were excluded if they: (i) included participants with medical or neurological conditions that independently affect sleep or MVPA profiles. (ii) Focused on preschool populations or adults over 18 years. (iii) Did not simultaneously examine overweight/obesity, sleep, and MVPA profiles. (iv) Were editorials, opinion articles, narrative reviews, systematic reviews, meta-analyses, protocols without results, or grey literature. (v) Presented incomplete or unverifiable results. (vi) Were conducted solely in hospital, community, or university settings outside of educational or family environments.

### 2.4 Search strategy

The search covered studies published in the databases PubMed, Scopus, Web of Science, and LILACS between 2002 and 2024, a period marked by the development of standardized assessment tools and conceptual frameworks addressing sleep, MVPA profiles, and obesity in pediatric populations. The search strategy followed the PCC framework, combining controlled vocabulary terms (MeSH and DeCS) with free-text keywords. We used Boolean operators and truncation symbols to improve both search sensitivity and specificity. We used the following terms according to PCC: Population: childhood obesity, child overweight, pediatric obesity. Concept: sleep quality, sleep-related behavioral patterns, sleep disorders, MVPA profiles, motor activity. Context: educational system, family environment. We combined these components with the Boolean operator AND, while linking synonyms within each component with OR. We tailored search equations for each database. We limited results to studies conducted in children and adolescents, published between 2002 and 2024, in English, Spanish, or Portuguese.

The following terms were selected: (“Adolescent Obesity” OR “Adolescent Overweight” OR “Child Obesity” OR “Childhood Obesity” OR “Childhood Onset Obesity” OR “Childhood Overweight” OR “Pediatric Obesity”) AND (“school-based physical activity” OR Exercise* OR “Physical Education and Training” OR “Motor Activity”) AND (“Sleep quality” OR “Sleep deprivation” OR “sleep disorders” OR “sleep hygiene” OR “sleep efficiency” OR “sleep duration”).

### 2.5 Study selection

We used the systematic review platform Rayyan (Rayyan Systems Inc.), which facilitated duplicate removal and independent screening. Two reviewers independently screened the titles and abstracts of the retrieved records. Reviewers then assessed potentially eligible studies by full-text review against the predefined PCC eligibility criteria. Reviewers resolved disagreements through discussion and, when necessary, consultation with a third senior reviewer. We identified 1,086 records across the selected databases. After removing 407 duplicate records, 679 records remained for title and abstract screening. We excluded 514 for not meeting the eligibility criteria, leaving 165 reports for full-text assessment. Of the 165 reports sought for retrieval, one full-text report could not be retrieved, leaving 164 reports for eligibility assessment. Following full-text evaluation, 118 reports were excluded because they did not meet the eligibility criteria. Ultimately, 46 studies met the eligibility criteria and were included in the final synthesis. The PRISMA flow diagram presents the study selection process (see Figure 1).

**Figure 1:**
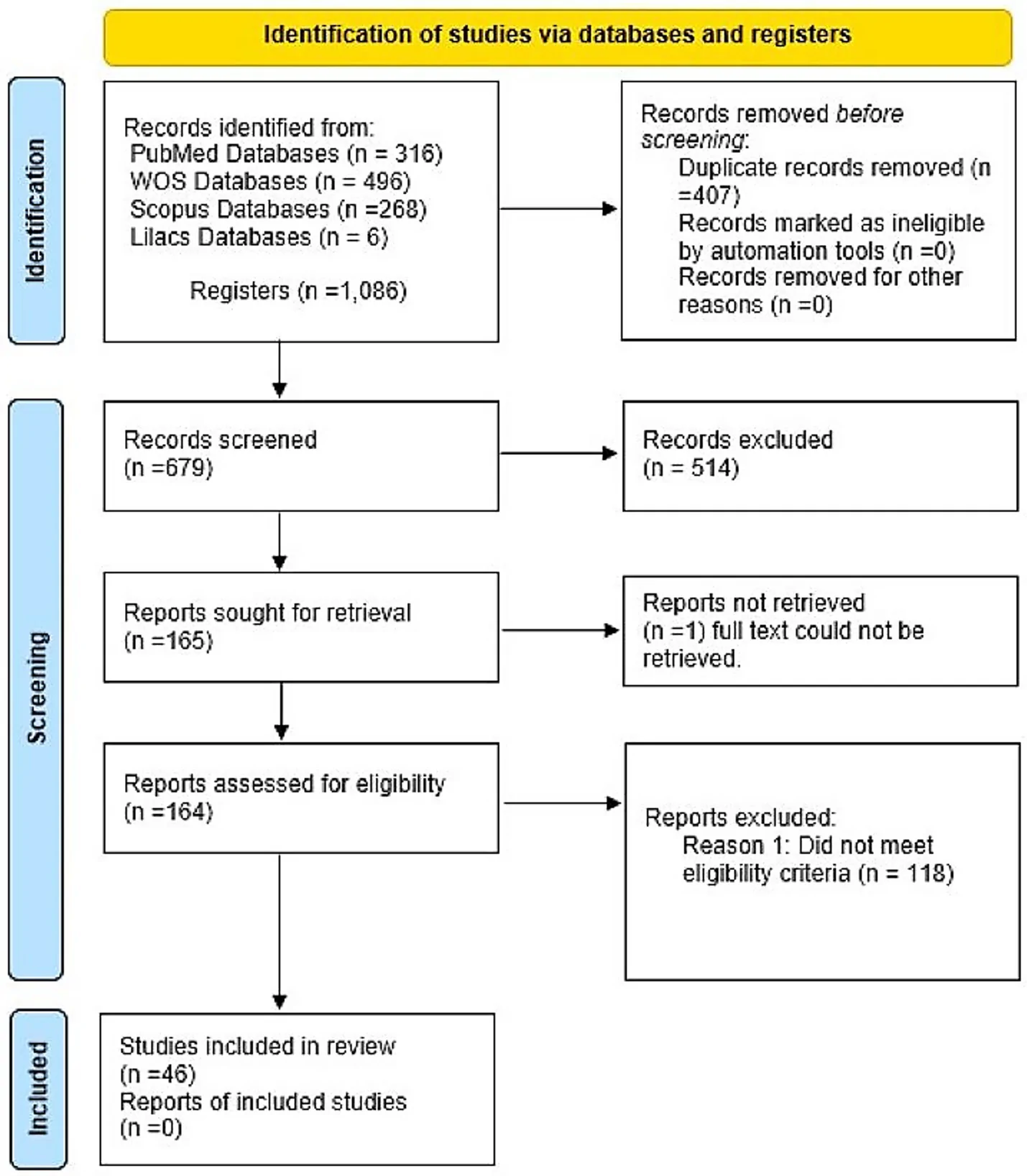
Selection process, including identification, screening, eligibility, and inclusion of studies.

### 2.6 Data extraction and synthesis

Two reviewers conducted data extraction independently and in duplicate using a standardized extraction matrix based on recommendations from the Joanna Briggs Institute (25). The following data were collected: (a) Author and publication year; (b) Country where the study was conducted; (c) Study design; (d) Context (family and/or school); (e) Sleep assessment tools; (f) Type of MVPA profiles or sedentary behavior evaluated; (g) Anthropometric classification criteria; (h) Main findings of the study. We resolved disagreements through discussion and consensus, involving a third reviewer when necessary. The results were synthesized using a descriptive narrative approach, organizing the evidence into three analytical categories: (a) methodological and demographic characteristics of the studies; (b) sleep quality dimensions associated with MVPA profiles; and (c) sleep dimensions associated with sedentary behavior, screen exposure, and broader behavioral patterns related to obesogenic lifestyles.

### 2.7 Ethical considerations

Because this review relied solely on secondary data from publicly available sources, it did not require ethical approval.

## 3 Results

### 3.1 General characteristics of the studies

Of the 46 included studies, the majority were conducted in North America, with 22 in the United States (47.8%) and 14 in Canada (30.4%). In South America, 6 were in Brazil, 2 in Argentina, 1 in Mexico, and 1 in Chile, accounting for almost one-third (Figure 2).

**Figure 2:**
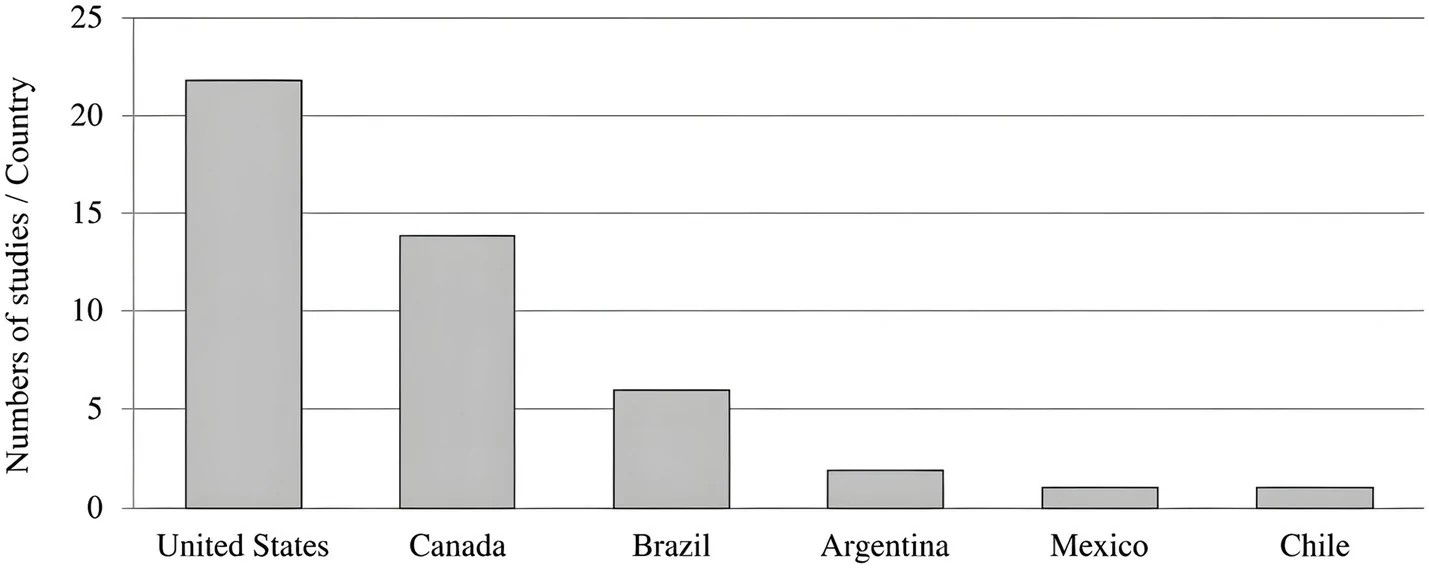
Most studies were conducted in the United States (47.8%) and Canada (30.4%), indicating a strong concentration of evidence in North America, while South American countries were underrepresented.

Cross-sectional observational designs were the most common (69.5%), while studies focusing on school- or family-based interventions accounted for 30.5% of the included studies (26–31). For behavioral assessment, 57% of studies used accelerometry to measure MVPA profiles, and 62% used standardized tools to evaluate sleep, including PSQI, actigraphy, or parental questionnaires (29, 32–34). Participants were primarily school-aged children aged 8 to 12 years, classified as overweight or obese based on BMI or age-adjusted percentiles (35–37). Studies differed methodologically and conceptually in how they assessed MVPA profiles and sleep. Most studies examined relationships among sleep, Moderate-to-Vigorous Physical Activity, sedentary behavior, and body-fat indicators. Univariate and multivariate analyses were the most commonly used analytical methods. Studies also frequently included variables related to family, school, and socioenvironmental factors (see Supplementary Table 1).

### 3.2 Movement behaviors and sleep dimensions

Studies using objective movement measurements consistently report that moderate-to-vigorous physical activity was associated with more favorable sleep outcomes, including longer sleep duration, greater sleep efficiency, lower fragmentation, and more regular sleep patterns (36, 38–43). Conversely, sedentary behavior and prolonged screen exposure, particularly before bedtime, are associated with poorer sleep indicators, including shorter sleep duration, reduced sleep continuity, daytime sleepiness, and lower subjective sleep satisfaction (27, 28, 31, 34, 40). Figure 3 conceptually summarizes the direction of these associations and illustrates how physical activity and sedentary behavior, assessed using a Moderate-to-Vigorous Physical Activity (MVPA) profile, can distinctly influence multiple dimensions of sleep quality in overweight or obese children and adolescents.

**Figure 3:**
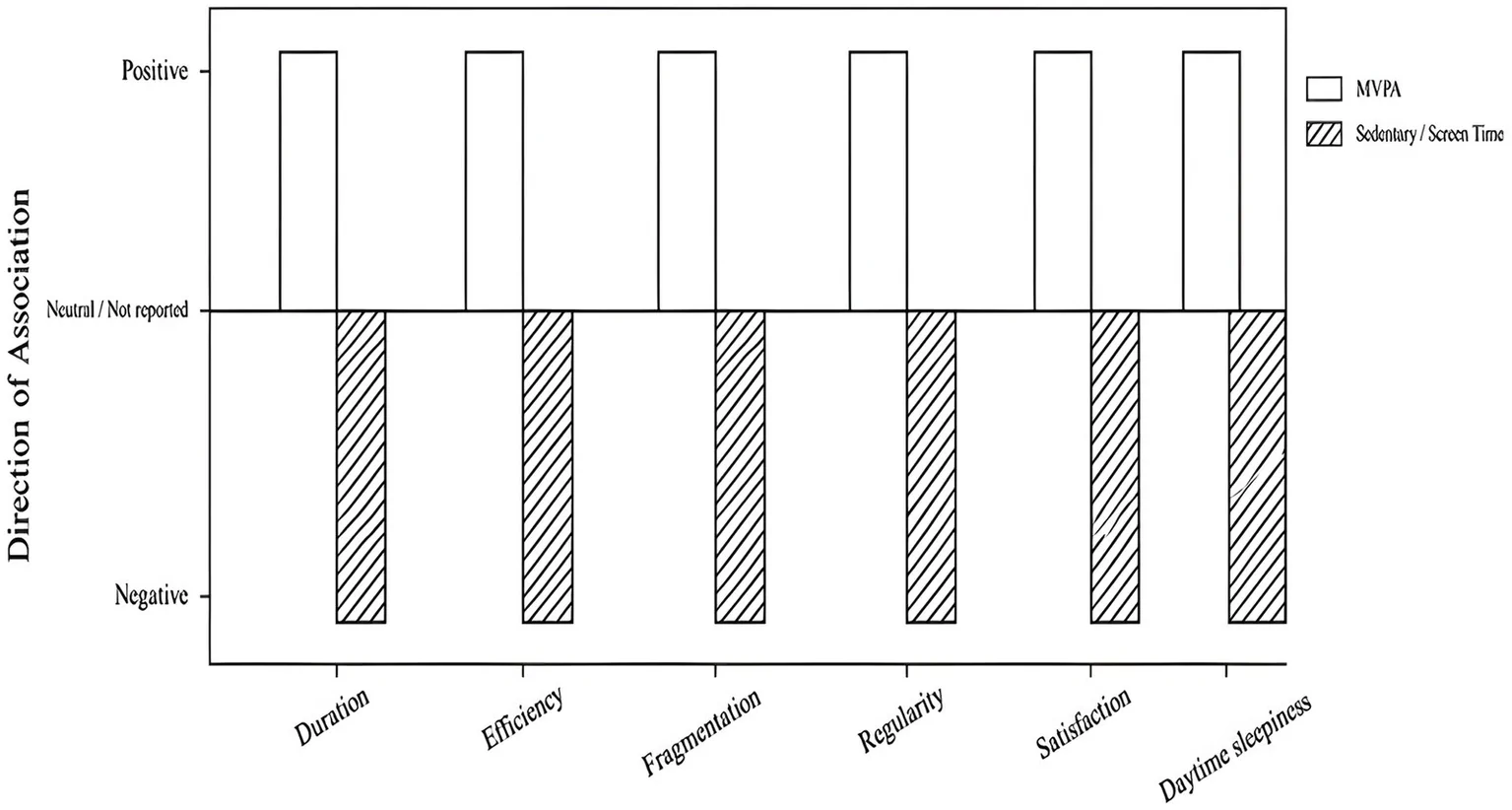
Conceptual direction of the associations between MVPA, sedentary behavior, and sleep dimensions.

The temporal distribution of the included studies suggests a growing scientific interest in the relationships between sleep quality, MVPA, and obesity-related health outcomes in pediatric populations. As shown in Figure 4, publication frequency increased substantially after 2014, with the highest concentration of studies between 2018 and 2024, reflecting growing recognition of sleep and movement behaviors as interconnected determinants of child health.

**Figure 4:**
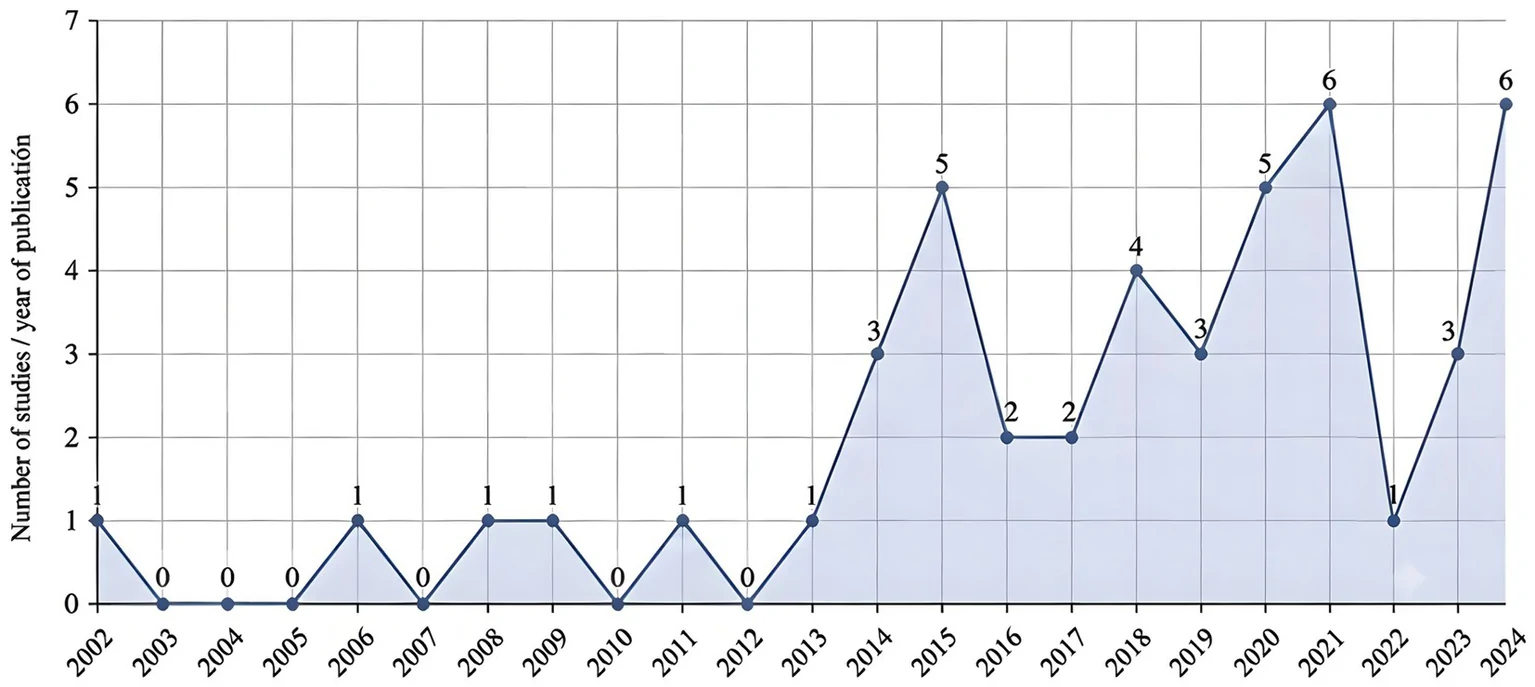
The figure shows a gradual increase in publications between 2002 and 2024, with a more pronounced rise from 2014 onwards, and the highest concentration of studies between 2020 and 2024. This pattern reflects the growing scientific interest in the interrelationships among sleep quality, movement behavior, sedentary lifestyles, and obesity in the pediatric population.

### 3.3 Behavioral patterns, sedentary routines, and obesity risk

Across the included studies, a moderate-to-vigorous physical activity profile was consistently associated with more favorable sleep outcomes (30, 35, 41, 44), particularly longer sleep-related behavioral patterns, greater sleep efficiency, and reduced sleep fragmentation among children and adolescents with overweight or obesity (45–48). Sedentary behavioral patterns, particularly prolonged screen exposure before bedtime, were consistently associated with poorer sleep outcomes and higher adiposity indicators (43, 49, 50). These associations mainly involved shorter sleep duration, reduced sleep satisfaction, and greater daytime sleepiness (39). In children who were overweight or obese, prolonged sedentary routines were also connected to irregular sleep schedules and difficulty falling asleep (28, 36, 51, 52).

### 3.4 Gaps in sleep assessment and multicomponent approaches

Although most studies included at least one sleep-related variable, sleep-related behavioral patterns were the most commonly reported dimension. Other aspects, such as sleep latency and daytime sleepiness, were assessed less frequently (29, 36, 53–56). This trend also appeared in studies using objective measurement tools, where sleep-related behavioral patterns were reported more often than other dimensions (48, 53, 54, 57). Only a small number of studies examined sleep, Moderate-to-Vigorous Physical Activity, and sedentary behavior simultaneously (29, 34, 42, 58). Most research looked at these factors separately. Studies that adopted a multicomponent approach identified distinct behavioral profiles and reported bidirectional relationships among MVPA profiles, sleep patterns, and sedentary behaviors in children with overweight or obesity (33, 34, 42, 45), except for one app-based study (59). Some also included measures related to chronobiology and physical literacy (48, 60).

## 4 Discussion

### 4.1 Sleep quality and movement behaviors in pediatric obesity

This exploratory review synthesized the available evidence on the relationship between sleep quality and 24-h movement behaviors in overweight or obese school-aged children in the Americas. In the reviewed studies, physical activity, sedentary behavior, and screen time were consistently associated with multiple dimensions of sleep, particularly duration, efficiency, regularity, and daytime sleepiness (30, 40–42). Similar patterns emerged in studies examining MVPA profiles in pediatric obesity, supporting the view that these behaviors should be understood as interdependent components of the 24-h movement behavior paradigm rather than isolated lifestyle factors (43, 61–63). These findings are especially relevant in the Americas, where childhood obesity often coexists with insufficient physical activity, prolonged sedentary behavior, and poor sleep patterns, contributing to clustered behavioral risk profiles (26, 64–66). A key finding of this review is that the observed relationship between sleep and movement behaviors remains limited by the incomplete and heterogeneous assessment of sleep quality (21, 53, 61, 67). Although sleep quality is widely recognized as a multidimensional construct encompassing duration, latency, efficiency, continuity, regularity, satisfaction, and daytime functioning (18, 22), most studies focus primarily on sleep duration and, less frequently, sleep efficiency (53, 55, 61, 67). Consequently, important dimensions of sleep remain underrepresented, limiting a more comprehensive understanding of how sleep interacts with physical activity and sedentary behavior in overweight or obese children (21, 67). This gap is consistent with the broader literature on pediatric obesity, which continues to prioritize sleep duration over other dimensions of sleep quality (7, 21, 67). Although objective tools such as accelerometry and actigraphy are increasingly used, considerable heterogeneity persists in sleep assessment protocols and analytical approaches (21, 22), limiting comparability between studies and potentially underestimating sleep’s broader role in pediatric obesity (7, 8, 18, 68). From this perspective, an incomplete assessment of sleep quality not only limits understanding of sleep-related outcomes but also hinders the identification of broader behavioral mechanisms associated with childhood obesity. While sleep quality and movement behaviors appear closely interconnected, behavioral self-regulation remains largely unexplored and may represent an important pathway linking these factors to obesity risk.

This methodological heterogeneity was particularly evident in sleep assessment. While sleep quality encompasses duration, latency, efficiency, continuity, regularity, subjective satisfaction, and daytime functioning (5, 17), most included studies focused primarily on sleep duration and assessed sleep efficiency less frequently (5, 21, 69, 70). Furthermore, researchers measured sleep outcomes using heterogeneous methods, including actigraphy, standardized questionnaires, parental reports, and self-reported sleep indicators (21, 54, 71). These approaches differ in their ability to capture objective and subjective dimensions of sleep and are not directly interchangeable (17, 21, 54). Consequently, interpret the apparent consistency of associations between sleep and movement behaviors with caution, as differences in measurement instruments, scoring procedures, observation periods, and sleep dimensions may have contributed to variability across studies (21, 54, 69, 71). Taken together, these findings suggest that sleep quality and movement behaviors should not be interpreted as isolated lifestyle factors, but rather as interconnected components influenced by broader family and school environments from a multilevel perspective. This contextual influence is supported by recent evidence showing that parents who met all three 24-h movement guidelines were more likely to have children who also met the combined recommendations (adjusted OR = 3.67, 95% CI: 1.14–11.76), highlighting parental modeling as a potentially relevant correlate of children’s movement behaviors (72).

Overall, the reviewed literature presents both methodological strengths and significant limitations. A major strength is the growing use of objective tools, such as accelerometry and actigraphy, which reduces reliance on self-reported or third-party-provided behavioral data and allows more precise characterization of movement and sleep patterns. Several longitudinal and multicomponent studies also provide evidence of temporal and potentially bidirectional relationships between sleep, physical activity, and sedentary behavior. However, these advantages are offset by the predominance of cross-sectional designs, heterogeneous assessment protocols, inconsistent operational definitions, and the frequent reduction of sleep quality to sleep duration alone. Consequently, the available literature provides reasonably consistent evidence of association, but remains less conclusive regarding causality, effect size, and the mechanisms underlying these relationships.

### 4.2 Sedentary behaviors, physical activity, and obesogenic behavior profiles

Another important finding was the interconnection between sleep, physical activity, and sedentary behavior. Rather than functioning as separate lifestyle factors, these behaviors were consistently interrelated across the reviewed studies, resulting in behavioral patterns associated with lower or higher obesity risk (31, 41, 45, 73). Similar relationships were reported in studies examining combinations of movement behaviors and weight-related outcomes, reinforcing the importance of considering these behaviors as an integrated rather than isolated system (42, 46, 62, 63). Children who were more physically active and less sedentary generally exhibited more favorable sleep indicators (30, 42, 43, 62). Conversely, prolonged sedentary behavior and increased screen time were consistently associated with shorter sleep duration, poorer sleep quality, and less healthy weight profiles (50, 51, 61, 74). This observation aligns with the growing 24-h movement behavior framework, which views sleep, physical activity, and sedentary time as interconnected components of daily time use (17, 21). From this perspective, incomplete sleep measurement not only impacts sleep research but also undermines our understanding of the broader behavioral profile associated with childhood excess weight.

Sedentary behavior, particularly screen exposure before bedtime, was consistently associated with worse sleep outcomes (29, 36, 42, 43). These associations included shorter sleep duration and reduced sleep continuity (30, 38), as well as increased daytime sleepiness and, in some studies, greater adiposity or less favorable eating habits (50, 74, 75). This pattern suggests a behavioral risk cycle in which nighttime screen exposure, sleep deprivation, and obesity-related behaviors reinforce each other. Existing evidence supports several plausible mechanisms, including delayed sleep onset, circadian misalignment, emotional overstimulation, and disrupted appetite regulation (12, 31, 38, 47). Although causal relationships are not firmly established, the consistency of these associations across multiple studies underscores the importance of addressing sedentary nighttime routines in prevention strategies.

Conversely, physical activity emerged as a potential protective factor. Across all reviewed studies, higher levels of physical activity, particularly moderate-to-vigorous physical activity, were consistently associated with better sleep markers and lower adiposity (41, 42, 48, 59). Similar findings were reported in studies assessing movement behavior patterns and obesity-related outcomes (59, 60, 74, 76). These observations align with international recommendations that recognize physical activity as a fundamental component of healthy daily routines in childhood (6, 18). However, evidence also suggests that these relationships are dynamic rather than strictly linear. Daily analyses in obese Latino children, for example, have demonstrated bidirectional associations between sleep and next-day movement behaviors, although the strength of these associations varies with activity intensity (19). Taken together, these findings indicate that sleep, physical activity, and sedentary behavior are best understood as interdependent behavioral profiles rather than independent lifestyle factors. This ecological-behavioral perspective supports the hypothesis that underlying behavioral self-regulation may partially explain how these behaviors interact over time, while also underscoring the need for further longitudinal and multicomponent studies to clarify their combined contribution to obesity risk. Heterogeneity was also observed in how physical activity and sedentary behavior were measured and analyzed (21, 30, 69). While several studies used accelerometry or other device-based measurements, others relied on questionnaires, parental reports, school records, or estimates of screen exposure (31, 46, 69, 71). Definitions and thresholds for moderate-to-vigorous physical activity, sedentary time, and adherence to movement guidelines also varied across studies (21, 30, 31). Furthermore, the predominance of cross-sectional designs limited the ability to determine the temporal direction, whereas longitudinal and intervention studies differed in follow-up duration, intervention content, and outcome evaluation (21, 30, 69). These methodological differences limit direct comparisons and suggest that the observed associations should not be interpreted as uniform effect estimates or as evidence of causality (21, 30).

### 4.3 Behavioral self-regulation and implications for health promotion

A key emerging perspective highlighted in this review is the potential role of behavioral self-regulation as a mechanism linking sleep quality, 24-h movement behaviors, and obesity risk. Although only a limited number of included studies explicitly assessed executive functions, the available evidence suggests that sleep deprivation and excessive sedentary behavior can impair inhibitory control, attentional regulation, and decision-making. In contrast, healthier sleep patterns and higher levels of physical activity may promote self-regulatory capacities associated with healthier behavioral choices (22, 23). While behavioral self-regulation has rarely been measured directly, the consistent associations observed in the reviewed studies suggest it may represent an important explanatory pathway warranting further longitudinal investigation.

Another important finding is the limited adoption of truly multicomponent approaches. Only a minority of studies simultaneously examined sleep, physical activity, and sedentary behavior (41, 42, 45, 46), despite evidence indicating that these behaviors are embedded within broader behavioral profiles shaped by family routines, school environments, and neighborhood characteristics (35, 44, 77, 78). Taken together, these findings support an ecological-behavioral perspective in which movement behaviors emerge through ongoing interactions between individual behavioral regulation and environmental contexts. From this perspective, interventions against childhood obesity should address sleep quality, physical activity, sedentary behavior, and their contextual determinants simultaneously, rather than treating them as isolated targets. For nurses and other child health professionals, this underscores the importance of family and community strategies that promote structured sleep routines, healthy movement behaviors, and reduced evening screen time (10, 29, 45, 59).

Interpreting these findings must account for the methodological heterogeneity of the available evidence. Studies differed substantially in how they assessed sleep quality and movement behaviors, used diverse measurement methods and analytical approaches, and were predominantly cross-sectional (21, 30, 53, 61). Furthermore, relatively few studies used integrated analytical frameworks that could simultaneously examine sleep, physical activity, sedentary behavior, and contextual determinants (19, 21, 33, 46). This methodological and conceptual fragmentation limits comparability between studies and underscores the need for harmonized multidimensional assessments and multicomponent longitudinal research.

Overall, this exploratory review suggests that sleep quality should no longer be considered an isolated behavioral characteristic or a mere secondary correlate of childhood obesity. Instead, sleep appears to be one component of an interconnected system of movement behaviors across 24 h, shaped by reciprocal interactions between behavioral regulation and environmental contexts. This ecological-behavioral perspective provides a coherent conceptual framework for interpreting the evidence synthesized in this review and can guide the design of future multicomponent interventions. This review also has limitations inherent to its scope, including restriction to studies conducted in the Americas, publication in English, Spanish, or Portuguese, and the exclusion of grey literature, which may have limited the breadth of the evidence identified. Based on this synthesis, Figure 5 summarizes the main relationships identified in the reviewed studies and proposes an integrative conceptual framework to guide future research.

**Figure 5:**
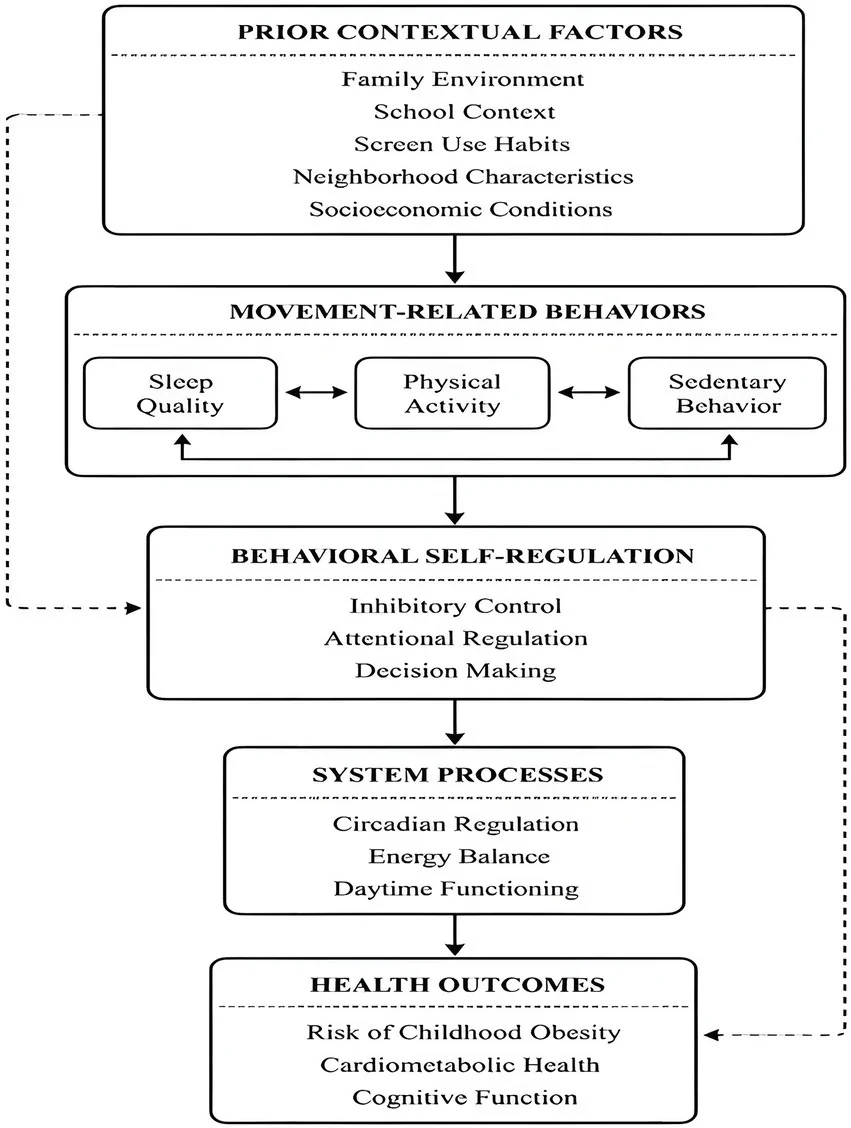
Sleep quality, physical activity, and sedentary behavior are conceptualized as interconnected behaviors influenced by family and school contexts, with behavioral self-regulation proposed as a potential mechanism linking these factors to obesity-related outcomes.

## 5 Conclusion

This exploratory review indicates that sleep quality, physical activity, and sedentary behavior are closely interconnected in overweight or obese school-aged children. However, the predominance of cross-sectional designs, variation between device-based and self-reported or third-party-reported measures, inconsistent definitions of movement behaviors, and the focus on sleep duration over other dimensions such as latency, continuity, regularity, satisfaction, and daytime functioning limit interpretation. Furthermore, only a minority of studies assessed sleep, physical activity, and sedentary behavior within an integrated, multicomponent framework. The findings suggest that these behaviors should not be considered in isolation, as they appear to form interconnected behavioral profiles associated with obesity risk. Moreover, behavioral self-regulation could be an important, though underexplored, mechanism linking sleep quality, movement-related behaviors, and obesity-related outcomes.

Future research should prioritize multidimensional sleep assessments, harmonized measurement strategies, and multicomponent longitudinal designs to examine how sleep, physical activity, sedentary behavior, and self-regulation processes interact in family and school contexts. Advances in this research could contribute to the development of more effective, context-specific strategies for childhood obesity prevention and health promotion.
